# Supplementary material for: Erratic and blood vessel-guided migration of astrocyte progenitors in the cerebral cortex
Source: Nat Commun. 2022 Nov 2;13:6571. doi: 10.1038/s41467-022-34184-x (PMC9630450; doi:10.1038/s41467-022-34184-x)
Supplement: Supplementary file 1 — Supplementary Information [file 41467_2022_34184_MOESM1_ESM.pdf]

Supplemental Information

Erratic and Blood Vessel-Guided Migration of Astrocyte Progenitors in the Cerebral Cortex

Tabata H. et al.

Supplementary Figure 1

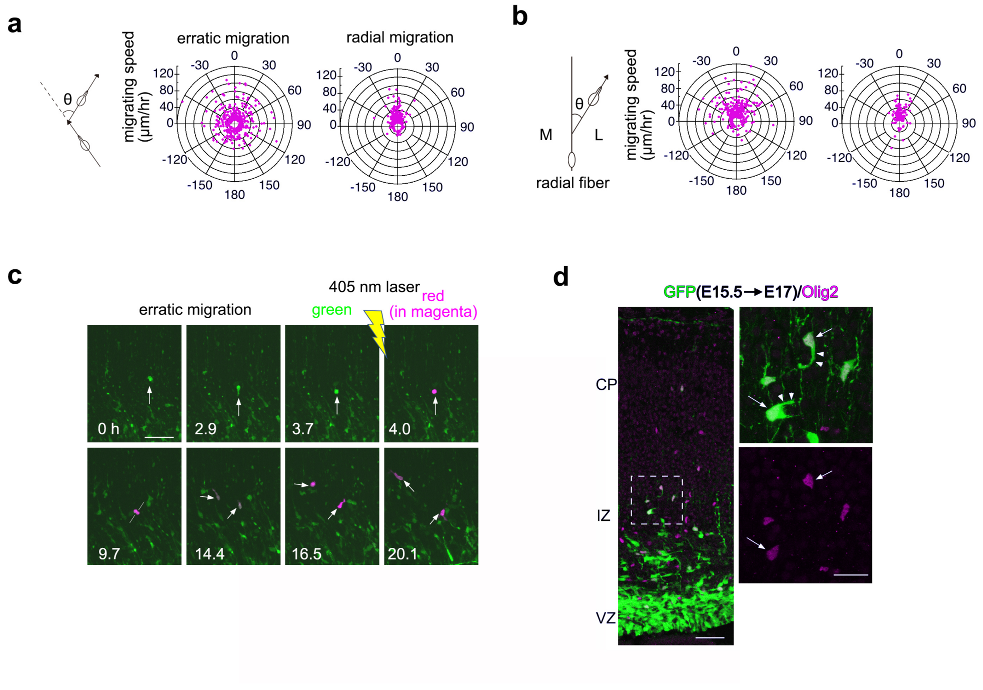

Supplementary Figure 1. Characterization of erratically migration cells.

a Directional changes in the movement (degree; positive values indicate a rightward turn in the right cerebral hemisphere) and migration speed (radius) in each frame. In radial migration, the directional changes between two consequent movements were mostly within 30º (75.8% ± 9.1%, average percentages of the movements whose directional changes are within 30º among all movements that individual cells made during the observation period, 10 cells in 4 slices, mean ± SD), whereas those during erratic migration showed a greater divergence (49.5% ± 10.5%, 11 cells in 4 slices, mean ± SD, two-sided unpaired t test with Welch's correction, radial vs erratic migration, P<0.0001). **b** Direction of movement relative to the radial fiber (degree; positive values indicate a lateral direction) and the migration speed (radius) in each frame. Although erratically migrating cells moved in various directions, the movement angle relative to the radial fibers and the speed was 12.8 ± 16.5º at 8.8 ± 1.3 μm/h (0º is parallel to the radial fibers toward the brain surface; data is for the mean of 11 cells in 4 slices ± SEM), indicating that they tended to move toward the brain surface, which is still divergent comparing to that of radial migration (1.2 ± 4.2º at 16.6 ± 1.6 μm/h, 8 cells in 4 slices, mean ± SD, two-sided unpaired t test with Welch's correction, radial vs erratic migration, P=0.0006). **c** (Related to Figure 1**d**) An example of photoconversion of erratically migrating cells with 405nm laser. The arrows point to a cell that was migrating in an erratic migration mode; this single cell was irradiated with a 405-nm laser between the "3.7 h" and "4.0 h" time frames, changing in the fluorescence from green to red (the red color is shown in magenta on these images). This cell divided at 9.7 hours, and the daughter cells also adopted an erratic migration mode. **d** $GFP^+/Olig2^+$ astrocyte progenitors represent the erratically migrating cell-like morphology in fixed brains. Embryos were electroporated with pCAG-EGFP (1 μg/μl) at E15.5 and were then fixed 36 hours later. Overall distribution of GFP+ cells is shown at left, and high magnification of the cells located in the superficial IZ to

bottom of CP is shown at right. GFP^+/$Olig2^+$ astrocyte progenitors begin to enter the superficial IZ and CP earlier than neurons. The vast majority of GFP^+ cells in these regions were $Olig2^+$ (right, 92.6%, 75 out of 81 GFP^+ cells from 6 brains). Their irregular morphology and the short leading process pointing in various directions represented the characteristics of erratically migrating cells (n=6 brains). Scale bars, 100 µm. 50 µm (**d**, left); 20 µm (**d**, right). Source data are provided as a Source Data file.

Supplementary Figure 2

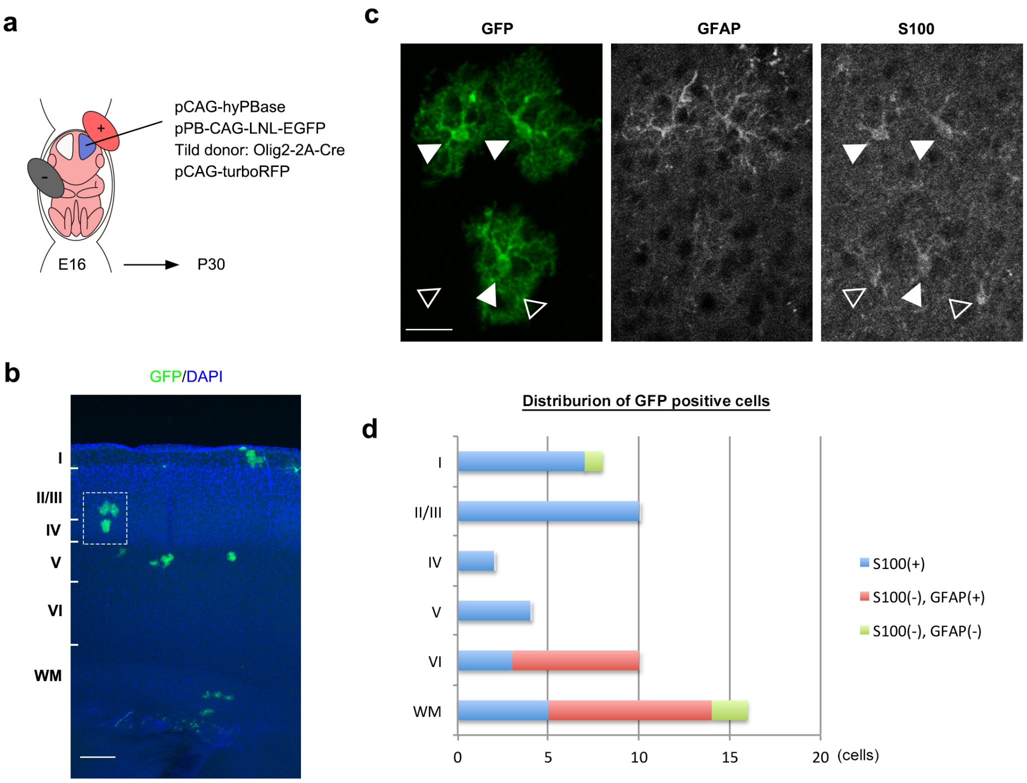

Supplementary Figure 2. Lineage tracing of Olig2-expressing cells derived from cortical VZ using the Tild-CRISPR method.

a 2A-Cre sequence was inserted into the *Olig2* gene *in utero* at E16 and the GFP-expressing cells were analyzed at P30. **b** One example of the brain containing GFP^+ cells. **c** High magnification view of the boxed area in (**b)** and its immunohistochemistry for GFAP and S100β. Closed and open arrowheads indicate GFP-positive and negative cells among S100β-positive astrocytes, respectively. **d** Histogram of GFP-positive cells in the CP (layer I~VI) and WM. They were classified into S100β-positive (protoplasmic astrocyte), S100β-negative but strongly GFAP-positive (fibrous astrocyte) or double negative cells. The double-positive cells were included in the S100β-positive cell column. Using the Tild-CRISPR method, fibrous astrocytes in the WM were also labeled, which was not observed when Cre activation was temporally induced with tamoxifen at E17 (Fig. 2h). These fibrous astrocytes were thought to be labeled due to the prolonged expression of Cre in later stages. The cortical VZ-derived Olig2-expressing cells mostly differentiated into either protoplasmic or fibrous astrocytes (94%, 50 GFP^+ cells from 5 brains). Scale bars, 200 μm **b**; 30 μm **c**. Source data are provided as a Source Data file.

Supplementary Figure 3

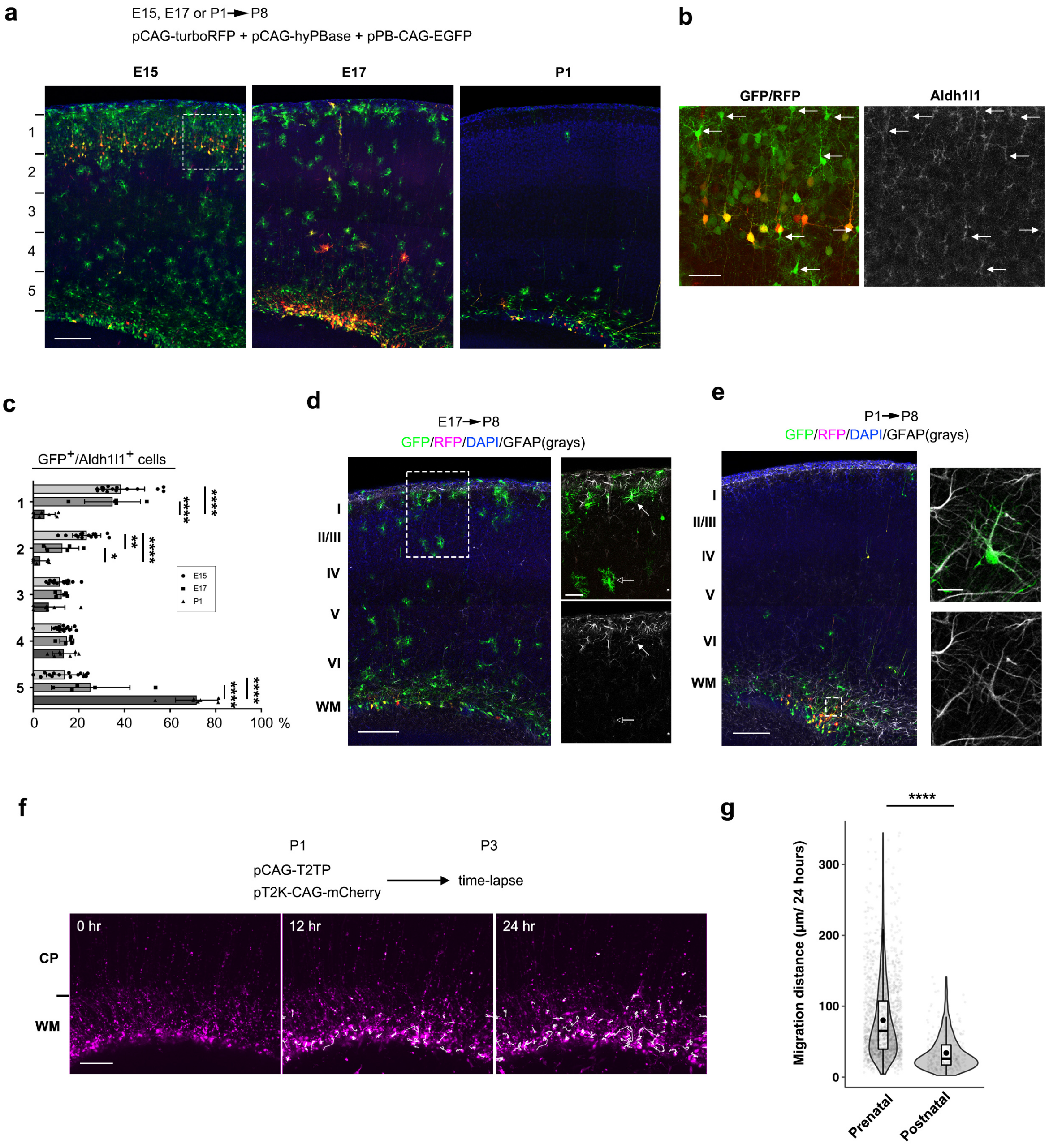

Supplementary Figure 3. Stage dependent positioning of astrocytes.

a The final positions of astrocytes electroporated at different timings. We electroporated E15, E17 or P1 embryos/pups with pPB-CAG-EGFP + pCAG-hyPBase + pCAG-turboRFP, which labels astrocytes with GFP and neurons with turboRFP, and fixed the brains at P8. **b** High magnification view of the boxed region in the left panel of (**a**). Many GFP^{+}/$Aldh1l1^{+}$ astrocytes (arrows) were found in layers II/III of the brain electroporated at E15. **c** Histogram of the GFP^{+}/$Aldh1l1^{+}$ cells in the brains electroporated at E15 (light gray bars, circle dots), E17 (middle gray bars, square dots) or P1 (dark gray bars, triangle dots) among 5 bins shown in the left side of (**a**). In the brains electroporated at P1, most of the GFP^{+}/$Aldh1l1^{+}$ astrocytes were located in the white matter (WM). In the case of E17, the GFP^{+}/$Aldh1l1^{+}$ astrocytes in the layer I were still produced, but the layer II/III and layer IV astrocytes, which are in bin2, were significantly reduced compared to the brains electroporated at E15 (one-way ANOVA followed by Tukey's multiple comparisons test, E15; 15 brains, E17; 5 brains, P1; 7 brains, average ± S.D., Bin1, E15 *vs.* P1, $P<0.0001$, E17 *vs.* P1, $P<0.0001$, bin 2; E15 *vs.* E17, $P=0.0045$, E15 *vs.* P1, $P<0.0001$, E17 *vs.* P1, $P=0.0231$, bin 5; E15 *vs.* P1, $P<0.0001$, E17 *vs.* P1, $P<0.0001$). (**d** and **e**) GFAP staining of the brains electroporated at E17 or P1. In the brains electroporated at E17, GFP^{+} layer I astrocytes were still labeled (5 out of 5 slices from 5 brains) (**d**). The high magnification view of the boxed region in the left panel is shown in the right. The strongly GFAP-positive layer I astrocytes (arrow) and GFAP-negative protoplasmic astrocytes (open arrow) could be observed. Postnatal VZ cells mainly produce fibrous astrocyte in the WM or deep layers of the CP (**e**). GFP^{+} cells were found mainly in the WM. High magnification view of the boxed region in the left panel is shown in right. The GFP^{+} cells in the WM highly expressed GFAP (arrows), and assumed fibrous astrocyte morphology (12 out of 12 slices from 12 brains). **f** Cells derived from postnatal VZ do not move extensively. P1 mouse pups were electroporated with Tol2 transposon vector system

(pCAG-T2TP + pT2K-CAG-mCherry], and subjected to time-lapse observations 2 days later. The mCherry-labeled cells (magenta) and their trajectories (white lines, cells that can be traced for more than 5 hours) were shown. Scale bar, 100 µm. See also Supplementary Movie 3. **g** The maximum distance achieved by each cell within 24 hour-observation period was represented in violin and box plots. Migration distances of E15 labeled and E18 observed cells that has been described in Fig.2 (turboRFP^{+}) were also analyzed in the same method for P1 labeled cells. The migration distance was significantly decreased in postnatally labeled cells compared to E15 labeled cells (Welch two sample t-test, 1748 E15-labeled cells vs 283 P1-labeled cells, ****P<0.0001). The definition of box plots is provided in the section of Statistical analysis in Methods. Scale bars, 200 µm (**a**, **d**-left, **e**-left), 100 µm (**f**), 50 µm (**b**, **d**-right), 20 µm (**e**-right). Source data are provided as a Source Data file.

Supplementary Figure 4

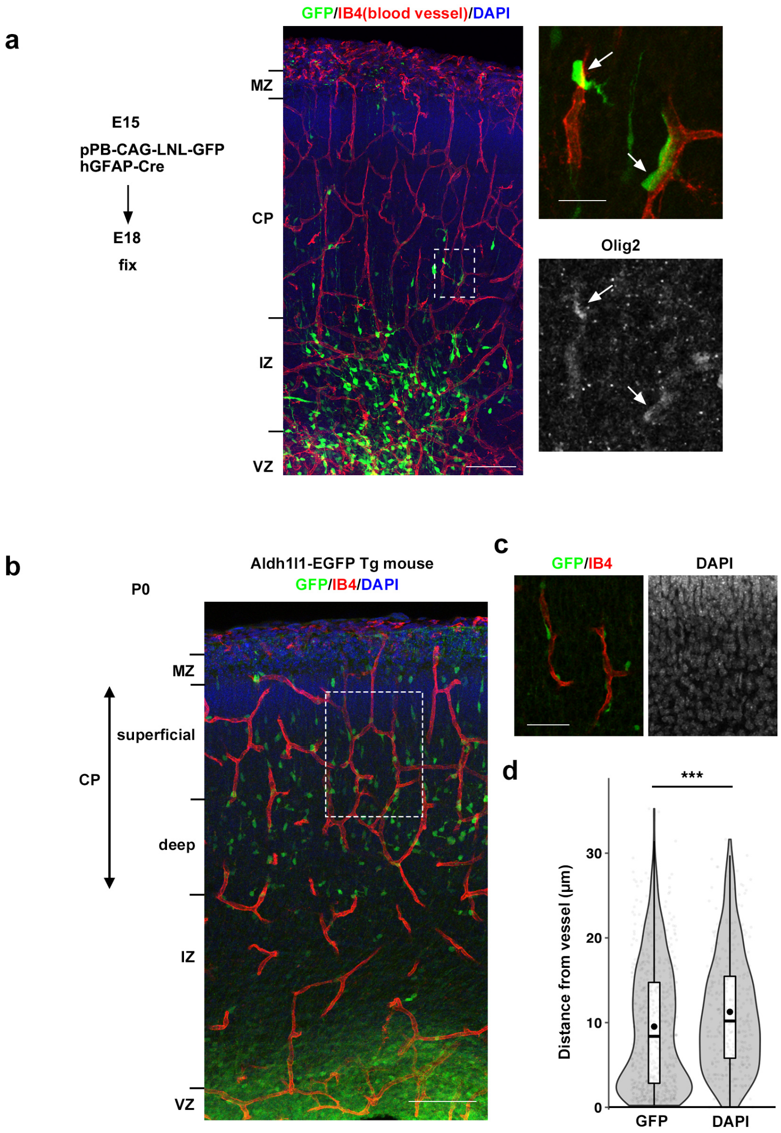

Supplementary Figure 4. Astrocyte progenitors are closely associated with blood vessels *in vivo*.

a Astrocyte progenitors labeled by *in utero* electroporation with a transposon vector were frequently associated with blood vessels. Human GFAP promoter driven Cre expression vector, hGFAP-Cre, and Cre-dependent GFP-expressing transposon vector, pPB-CAG-LNL-GFP, were electroporated at E15 and the brains were fixed 3 days later. The overview is shown on the left, and the high magnification view of the boxed region is shown on the right. Double-positive cells for GFP and Olig2, a marker for glial progenitors (pointed by arrows), were frequently associated with blood vessels stained with isolectin B4 (IB4, red) (71.3%, 57 out of 80 GFP^+ cells in the CP from 4 brains). (**b**-**d**) Genetically labeled astrocyte progenitors were also associated with blood vessels. The fixed P0 Aldh1l1-GFP mouse brains, in which GFP is expressed in astrocyte progenitors, were stained with IB4 (red), and the relationships with blood vessels were investigated. A single Z plane of the boxed region of (**b**) were shown in (**c**). GFP (green) and IB4 (red) channels and DAPI (grays) channel were separately presented. The distances between GFP^+ cells in the superficial CP and blood vessels were measured, and represented in violin and box plots (**d**). The GFP^+ cells were significantly closer to blood vessels than all cells stained by DAPI (Welch Two Sample t-test, ***$p< 0.001$, 639 GFP^+ cells vs 339 DAPI sained cells). The definition of box plots is provided in the section of Statistical analysis in Methods. Scale bars, 200 µm (**a**-left, **b**), 20 µm (**a**-right), 50 µm (**c**). Source data are provided as a Source Data file.

Supplementary Figure 5

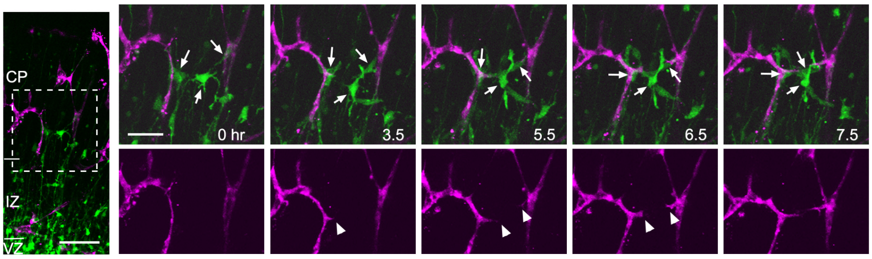

Supplementary Figure 5. Astrocyte progenitors support blood vessel branch formation. An example of blood vessel branch formation supported by astrocyte progenitors other than Figure 3F in slice culture. E15 flt1-DsRed mouse embryos were electroporated with pCAG-LNL-EGFP + pCAG-LNL-LynEGFP + Nestin-Cre and subjected to time-lapse observations next day. The time-lapse images in the boxed regions of the left panels are shown on the right. Three astrocyte progenitors (arrows) formed a bridge between two adjacent blood vessels, and then both blood vessels extended a process (arrowhead) along the astrocyte progenitors. Scale bars, 100 µm (left), 50 µm (right). See also Supplementary Movie 7.

Supplementary Figure 6

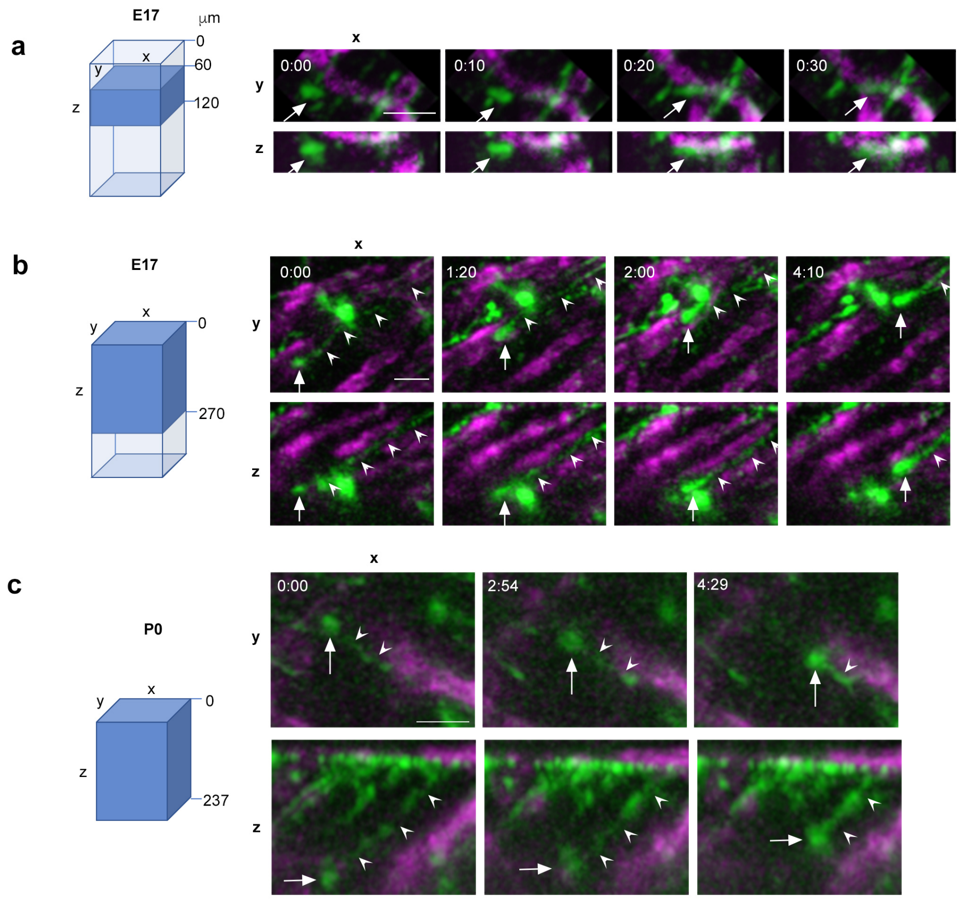

Supplementary Figure 6. Two-photon live imaging of astrocyte progenitors.

Embryos of flt1-DsRed mice were electroporated with the rDIO system and employed to *in vivo* imaging at E15 (**a**, **b**) or P0 (**c**). **a** One GFP positive cell (arrow) moved in the vicinity of a blood vessel, attached it, and migrated along it. See also Supplementary Movie 10. **b** Somal translocation mode observed in a living embryo. A GFP-positive cell (arrow) extending a long ascending process (arrowheads) to the brain surface retracted the process to elevate the cell body. See also Supplementary Movie 13. **c** Somal translocation of a GFP-positive cell (arrow) extending a long ascending process (arrowhead) observed in the intact P0 brain. See also Supplementary Movie 14. Scale bars: 20 µm.

Supplementary Figure 7

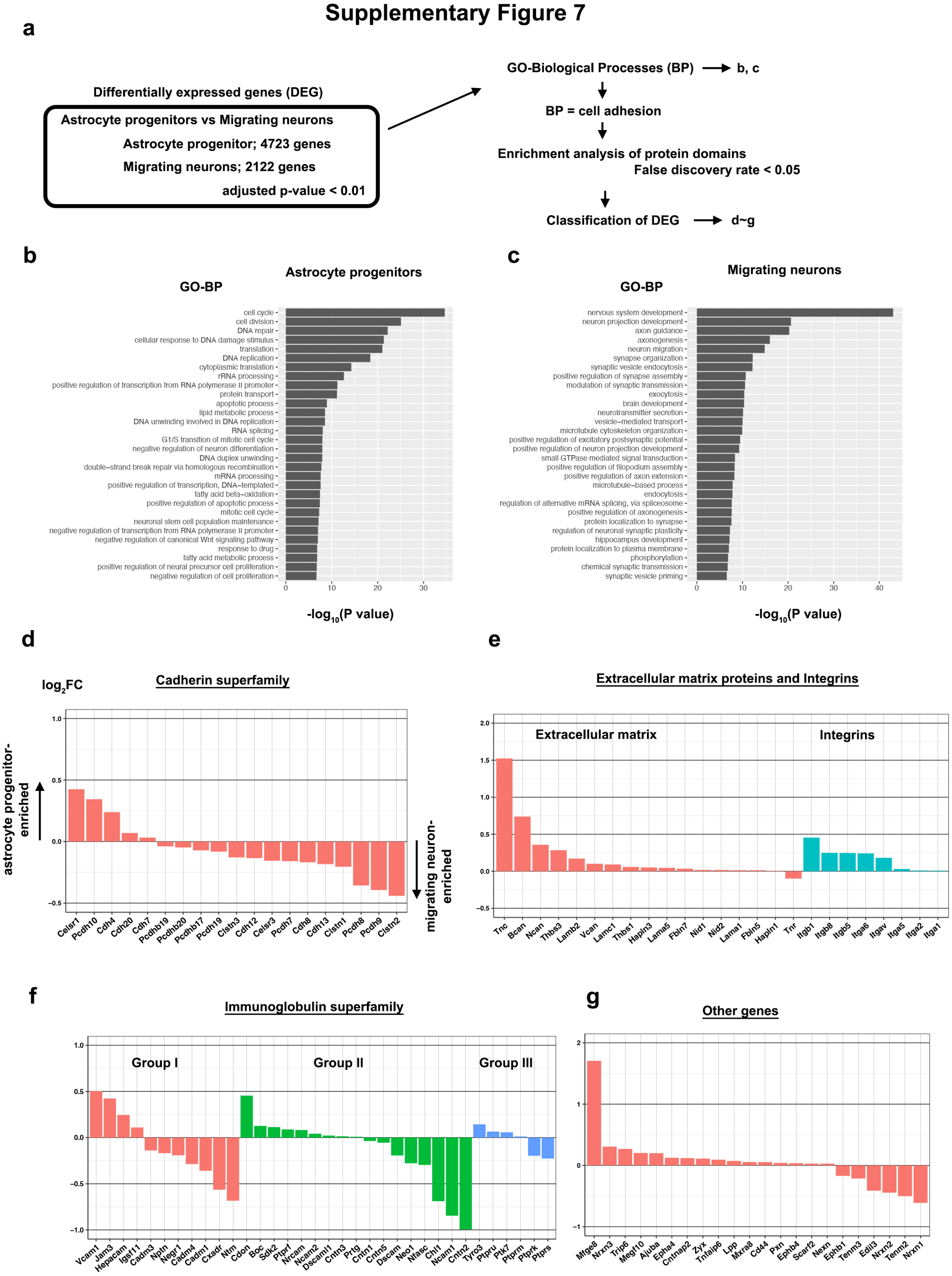

Supplementary Figure 7. Biological processes and molecular characteristics of differentially expressed genes between astrocyte progenitors and migrating neurons.

a The strategy for gene ontology (GO) analysis of differentially expressed genes (DEGs). We identified 4723 astrocyte progenitor-enriched DEGs and 2122 migrating neuron-enriched DEGs, and performed GO (biological processes, BP) enrichment analysis using DAVID bioinformatics resources for each gene set (**b** and **c**). We selected cell adhesion molecules (BP=cell adhesion), and classified them into gene families based on molecular structures (D-G). (**b** and **c**) GO (BP) enrichment analysis of astrocyte progenitor-enriched DEGs **b** and migrating neuron-enriched DEGs (C) (top-30 GO in P value for enrichment, which is generated by DAVID website in default settings; one-sided Fisher's Exact test). (D-G) DEGs annotated as "cell adhesion" were selected and were subjected into "protein domain" enrichment analysis. Genes including domains whose false discovery rate is less than 0.05 were taken into consideration. The Y-axis indicates the fold change (FC) of expression (astrocyte progenitors per migrating neurons). Source data are provided as a Source Data file.

Supplementary Figure 8

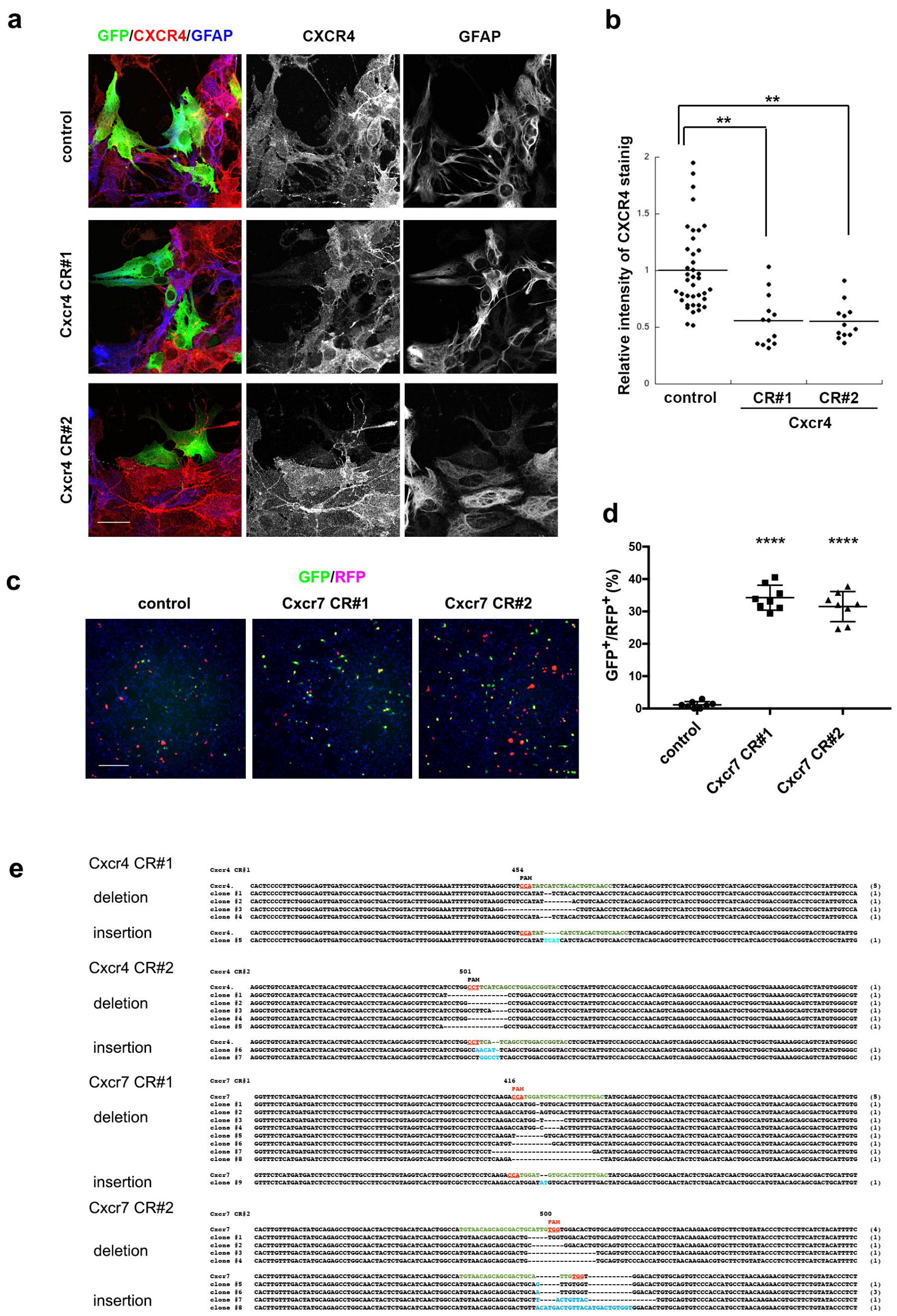

Supplementary Figure 8. Validation of CRISPR vectors for Cxcr4 and Cxcr7.

a Immunocytochemistry for endogenous Cxcr4 and GFAP of primary astrocytes transfected with two independent CRISPR vectors for Cxcr4. E16 mouse embryos were electroporated with CRISPR vectors, Cxcr4 CR#1 or CR#2, together with transposon EGFP expression vectors, pT2K-CAG-EGFP + pCAG-T2TP. On the next day, the transfected brains were taken out and cultivated *in vitro* for 4 days before the immunostaining. **b** Relative intensities of Cxcr4 staining in $GFP^{+}/GFAP^{+}$ cells against controls were evaluated (two-sided Dunnett's multiple comparisons test, control vs Cxcr4 CR#1, $P < 0.0001$, control vs Cxcr4 CR#2, $P<0.0001$, n=39 control, n=13 Cxcr4 CR#1, n=12 Cxcr4 CR#2). **c** Validation of CRISPR vectors for Cxcr7 by using a pCAG-EGxxFP vector. Two CRISPR vectors against distinct target sequences of Cxcr7 were constructed and their efficiencies were evaluated by EGFP fluorescence using the pCAG-EGxxFP vector system (see Methods). COS7 cells were co-transfected with CRISPR vectors, corresponding pCAG-EGxxFP-Cxcr7, and pCAG-mCherry (RFP) vectors. GFP and mCherry expressions were observed two days later. **d** GFP^{+} rate in $mCherry^{+}$ cells in randomly selected objective fields were calculated (two-sided Dunnett's multiple comparisons test, control vs Cxcr7 CR#1, $P < 0.0001$, control vs Cxcr7 CR#2, $P<0.0001$, 8 objective fields/2 dishes/group, Data are presented as mean values +/- SD). **e** CRISPR vector-mediated mutations in the *Cxcr4* and *Cxcr7* locus. Genomic sequences of *Cxcr4* and *Cxcr7* loci after transfection of two independent CRISPR vectors are shown. The target loci were amplified by PCR and cloned into a plasmid vector, and the amplicons were sequenced. The wild-type sequence of mouse *Cxcr4* and *Cxcr7* genes are shown in the first row, and the mutated sequences of deletion and insertion we observed are shown below. The observed numbers of each mutation or non-mutation are shown on the right end. The PAM sequence and the target sequence are highlighted in red and green, respectively. The insertions are highlighted in blue. The indel ratios of Cxcr4 CR#1 and CR#2, Cxcr7 CR#1

and CR#2 were 50% (5/10), 88% (7/8), 64% (9/14) and 71% (10/14), respectively. Scale bar, (A) 30 μm, (C) 200 μm. Source data are provided as a Source Data file.

Supplementary Figure 9

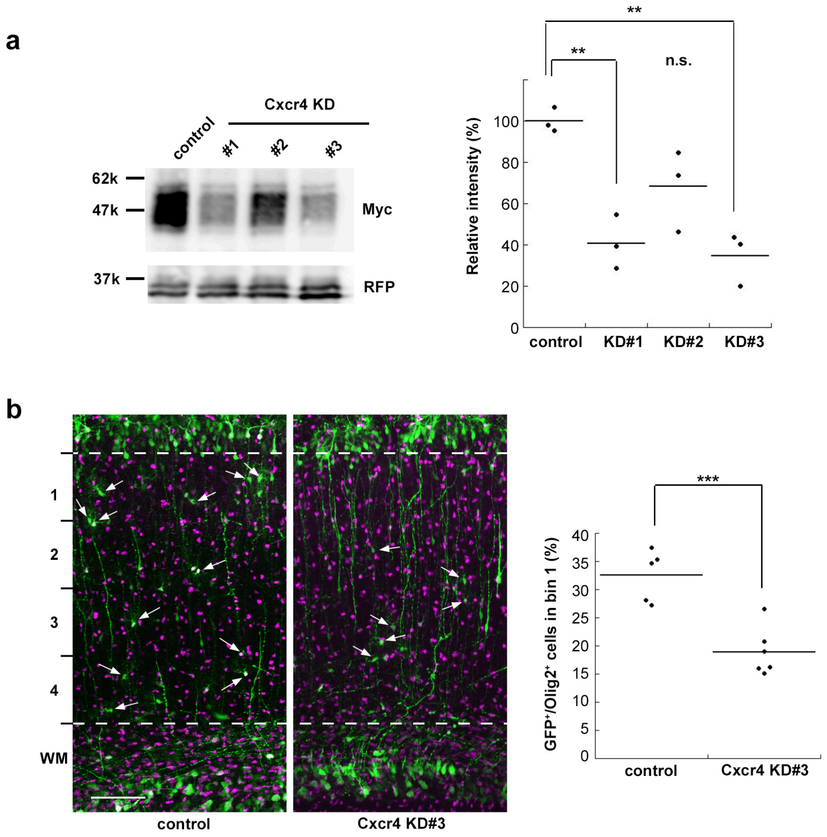

Supplementary Figure 9. Knockdown of Cxcr4 disrupt the positioning of protoplasmic astrocytes in the CP.

a Three independent miRNA expressing transposon vectors against mouse Cxcr4 were constructed (Cxcr4 KD#1~3). COS7 cells were transfected with these miRNA vectors together with Myc-tagged mouse Cxcr4 expression vector (pCAG-Myc-CxcR4) and mCherry expression vector (pCAG-mCherry). After 48h, cells were harvested and subjected to Western blotting with anti-Myc. Anti-RFP was used as a loading control (left). The relative intensities of Myc to control (miRNA vector for lacZ) are shown on the right (averages of three independent experiments). miR-CXCR4 #1 and #3 were effective (two-sided Dunnett's multiple comparisons test, control vs Cxcr4 KD#1 P=0.002, control vs Cxcr4 KD#3 P=0.0011). **b** E16 brains were electroporated with Cxcr4 KD#3 + pCAG-T2TP. The brains were fixed at P4 and the GFP^{+}/Olig2^{+} cells in the 4 bins in the CP were counted. The percentage of the cells found in bin 1 was significantly reduced (two-sided Student's t-test, control *vs.* Cxcr4 KD#3, P=0.006, n=5 control, n=6 Cxcr4 KD#3). Scale bar, 100 µm. Source data are provided as a Source Data file.

Supplementary Figure 10

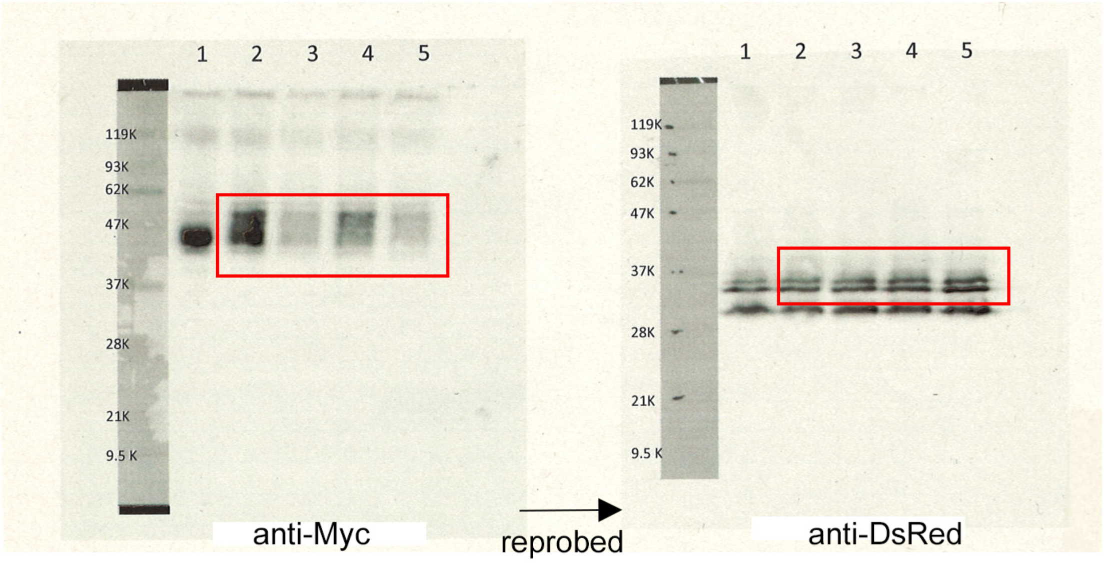

Supplementary Figure 10. Uncropped images of Western blots for Supplementary Figure 9a. The sample for lane #1 was from cells transfected with Cxcr4 expression vector but without KD vectors.
