# Supplementary material for: Erratic and blood vessel-guided migration of astrocyte progenitors in the cerebral cortex
Source: Nat Commun. 2022 Nov 2;13:6571. doi: 10.1038/s41467-022-34184-x (PMC9630450; doi:10.1038/s41467-022-34184-x)
Supplement: Supplementary file 3 — Description of Additional Supplementary Files [file 41467_2022_34184_MOESM3_ESM.pdf]

Description of Additional Supplementary Files

Supplementary Movie 1 Movie version of Fig. 1a

Supplementary Movie 2 Movie version of Fig. 1g

Supplementary Movie 3 Movie version of Supplementary Fig.3f

Supplementary Movie 4 Movie version of Fig. 3c

Supplementary Movie 5 Movie version of Fig. 3d

Supplementary Movie 6 Movie version of Fig. 3f

Supplementary Movie 7 Movie version of Supplementary Fig.5

Supplementary Movie 8 Movie version of Fig. 4b

Supplementary Movie 9 Movie version of Fig. 4c

Supplementary Movie 10 Movie version of Supplementary Fig.6a

Supplementary Movie 11 Movie version of Fig. 4d

Supplementary Movie 12 Movie version of Fig. 4e

Supplementary Movie 13 Movie version of Supplementary Fig.6b

Supplementary Movie 14 Movie version of Supplementary Fig.6c

Supplementary Movie 15 Movie version of Fig. 6c

Supplementary Movie 16 Movie version of Fig. 6d
