# Supplementary material for: Erratic and blood vessel-guided migration of astrocyte progenitors in the cerebral cortex
Source: Nat Commun. 2022 Nov 2;13:6571. doi: 10.1038/s41467-022-34184-x (PMC9630450; doi:10.1038/s41467-022-34184-x)
Supplement: Supplementary file 20 — Reporting Summary [file 41467_2022_34184_MOESM20_ESM.pdf]

nature portfolio

Corresponding author(s): Hidenori Tabata and Kazunori Nakajima

Last updated by author(s): Oct 9, 2022

Reporting Summary

Nature Portfolio wishes to improve the reproducibility of the work that we publish. This form provides structure for consistency and transparency in reporting. For further information on Nature Portfolio policies, see our Editorial Policies and the Editorial Policy Checklist.

Statistics

For all statistical analyses, confirm that the following items are present in the figure legend, table legend, main text, or Methods section.

n/a	Confirmed	
☐	☒	The exact sample size (*n*) for each experimental group/condition, given as a discrete number and unit of measurement
☐	☒	A statement on whether measurements were taken from distinct samples or whether the same sample was measured repeatedly
☐	☒	The statistical test(s) used AND whether they are one- or two-sided<br>*Only common tests should be described solely by name; describe more complex techniques in the Methods section.*
☐	☒	A description of all covariates tested
☐	☒	A description of any assumptions or corrections, such as tests of normality and adjustment for multiple comparisons
☐	☒	A full description of the statistical parameters including central tendency (e.g. means) or other basic estimates (e.g. regression coefficient) AND variation (e.g. standard deviation) or associated estimates of uncertainty (e.g. confidence intervals)
☐	☒	For null hypothesis testing, the test statistic (e.g. *F*, *t*, *r*) with confidence intervals, effect sizes, degrees of freedom and *P* value noted<br>*Give P values as exact values whenever suitable.*
☒	☐	For Bayesian analysis, information on the choice of priors and Markov chain Monte Carlo settings
☒	☐	For hierarchical and complex designs, identification of the appropriate level for tests and full reporting of outcomes
☒	☐	Estimates of effect sizes (e.g. Cohen's *d*, Pearson's *r*), indicating how they were calculated

Our web collection on statistics for biologists contains articles on many of the points above.

Software and code

Policy information about availability of computer code

Data collection	Time-lapse observations of living cells in brain slices or cultured cells on Matrigel were performed using Olympus FV1000.<br>Confocal images after immunohistochemistry were acquired using Olympus FV1000 (Fig. 1e, Supplementray Fig. 2c, 2b, 8a, 9b) or Zeiss LSM880 (the others).<br>Two-photon live imaging was performed using Nikon A1R MP.<br>Chemiluminescence images of Western blotting were captured using LAS4000 mini (GE Healthcare).<br>Flow cytometry was performed with CytoFlex S (Beckman Coulter).
Data analysis	Time-lapse image files (XYZT) were combined into movie files using 4D-Viewer software version 8.0.0 (Ratoc System Engineering).<br>Confocal images (Olympus oib files, or Zeiss czi files) and Western blotting data (gel files) were processed with ImageJ software version 4.0.93 (NIH).<br>For manual tracking of moving cells (Fig. 1a, 1b, 2a, 6d, Supplementary Fig. 1a, 1b), MTrackJ plug-in version 1.5.1 for ImageJ software was used. For automatic tracing of moving cells (Fig. 1g, 1h, 3e, 6c, Supplementary Fig.3f), TrackMate plug-in version 7.9.2 for ImageJ was used.<br>Two photon time-lapse Images were analyzed with MATLAB software version R2014a and ImageJ software version 4.0.93.<br>Single-cell RNA-seq data were analyzed using Cell Ranger version 1.3.0 protocol.<br>Seurat package (version 3.2.2) in R was used to analyze Single-cell RNA-seq data.<br>Astrocytes or astrocyte progenitors were automatically identified as GFP positive and Aldh1l1 positive (Fig. 6b, 7d, 8c) or RFP positive and Aldh1l1 positive (Fig. 8f), using Image Calculator function and 3D maxima finder plug-in version 4.0.93 for ImageJ software .<br>The 3 dimensional distances from glial progenitor cells to blood vessels were analyzed with 3D Object Counter and 3D RoiManager plug-ins version 4.0.93 for ImageJ (Fig. 6b, 7d, Supplementary Fig. 4d).<br>The relative positions of GFP or RFP positive/Aldh1l1 positive astrocytes (detected as mentioned above) to the upper and lower border of the CP (the borders were manually determined) were automatically measured using 3D RoiManager plug-in version 4.0.93 (Fig. 8c, 8f,

Supplementary Fig. c).
Statistical analyses were performed with Prism version 7 software (GraphPad) or R statistical package version 4.0.0. Violin and box plots were drawn by ggplot2 R package version 3.3.5.
Flow cytometry data were analyzed with FlowJo software version 10.8.1 (BD Biosciences).

For manuscripts utilizing custom algorithms or software that are central to the research but not yet described in published literature, software must be made available to editors and reviewers. We strongly encourage code deposition in a community repository (e.g. GitHub). See the Nature Portfolio guidelines for submitting code & software for further information.

Data

Policy information about availability of data

All manuscripts must include a data availability statement. This statement should provide the following information, where applicable:

- Accession codes, unique identifiers, or web links for publicly available datasets
- A description of any restrictions on data availability
- For clinical datasets or third party data, please ensure that the statement adheres to our policy

The all data showing in this study are available from Source Data file. All custom codes used in this study are available from the corresponding authors upon request. We do not have data to be archived, such as sequence data, or low data of single cell RNA-seq.

Human research participants

Policy information about studies involving human research participants and Sex and Gender in Research.

Reporting on sex and gender	not applicable
Population characteristics	not applicable
Recruitment	not applicable
Ethics oversight	not applicable

Note that full information on the approval of the study protocol must also be provided in the manuscript.

Field-specific reporting

Please select the one below that is the best fit for your research. If you are not sure, read the appropriate sections before making your selection.

☒ Life sciences ☐ Behavioural & social sciences ☐ Ecological, evolutionary & environmental sciences

For a reference copy of the document with all sections, see nature.com/documents/nr-reporting-summary-flat.pdf

Life sciences study design

All studies must disclose on these points even when the disclosure is negative.

Sample size	No statistical methods were applied to predetermine sample size. Three slices /brain x at least 7 brains/ (condition or group) were analyzed for statistical analyses of the distances from astrocyte progenitors to blood vessels and the distributions of astrocytes. We usually electroporated one experimental vector to 5~7 embryos, and the control vector to 3~4 embryos in the same litter. Typically, 4~6 experimental and 2~3 control brains are obtained, and they are enough for us to know the tendency. We repeated the same injection at least 2 times and confirm the reproducibility. In all other experiments (Western blots and in vitro culture), we conducted at least two biologically independent experiments to confirm the same tendency. With regards to the two-photon in vivo imaging, we provide images form one E17 embryo and one P0 pup that survived until the end of observations (monitored by circulation of blood). In these observations, we just confirmed the behaviors of astrocyte progenitors in the living mouse embryo/pup, and did not conduct statistical analysis.
Data exclusions	For statistic analyses of the distances to blood vessels and the position in the CP, the brains with low transfection efficiency (less than 20 cells/slice) were excluded from the analyses.<br>As to two-photon imaging, the data from embryos or pups died during the observations were excluded.
Replication	All statistic analyses were done from at least two independent experiments, and we confirmed the reproducibility.
Randomization	Embryos to be introduced with control vectors were randomly selected in the same litter in each in utero electroporation. Other than in utero electroporation experiments, cell lines and primary cultures are thought to be homogeneous and the bias among experimental groups can be ignored.
Blinding	We carried out the computer assisted measurements of the distances from the astrocyte progenitors to blood vessels (Fig.6b, 7d, Supplementary Fig. 4d), the relative positions of astrocytes in the CP (Fig. 8c, 8f, Supplementary Fig.3c), and the migration trajectories (Fig.1g,

1h, 3e, 6c, Supplementary Fig.3g).
When automated counting or tracing was not applicable, the investigators blinded to sample IDs performed evaluations.

Reporting for specific materials, systems and methods

We require information from authors about some types of materials, experimental systems and methods used in many studies. Here, indicate whether each material, system or method listed is relevant to your study. If you are not sure if a list item applies to your research, read the appropriate section before selecting a response.

Materials & experimental systems

n/a	Involved in the study
☐	☒ Antibodies
☐	☒ Eukaryotic cell lines
☒	☐ Palaeontology and archaeology
☐	☒ Animals and other organisms
☒	☐ Clinical data
☒	☐ Dual use research of concern

Methods

n/a	Involved in the study
☒	☐ ChIP-seq
☒	☐ Flow cytometry
☒	☐ MRI-based neuroimaging

Antibodies

Antibodies used

Primary antibodies:
chicken anti-GFP (Aves, GFP1010) (IHC 1:1000)
rabbit anti-GFP (MBL, #598) (IHC 1:1000)
rabbit anti-RFP (Rockland, #600-401-379) (IHC 1:1000, WB 1:500)
mouse anti-GFAP (Sigma, G3893, clone G-A-5) (IHC 1:500, ICC 1:1000)
rabbit anti-GFAP (Dako, ZO334) (ICC 1:400)
mouse anti-β-tubulin III (BioLegend/Covance, 801213, TuJ1) (ICC 1:1000)
rabbit anti-Myc (Mizutani et al. https://doi.org/10.1515/hsz-2012-0258) (WB 1:1000)
This anti-Myc antibody is generated in our laboratory (Prof K. Nagata's lab in Department of Molecular Neurobiology, Institute for Developmental Research, Aichi Developmental Disability Center).
goat anti-Olig2 (R&D, AF2418) (IHC 1:300)
goat anti-S100β (R&D, AF1820) (IHC 1:200)
goat anti-GSTπ(LifeSpan Biosciences, LS-B2376)(IHC 1:300)
rabbit ant-Aldh1l1 (Abcam, ab87117)(IHC 1:1000)
rabbit anti-Cxcr4 (Abcam, ab124824, clone UMB2) (ICC 1:300)
mouse anti-β-Actin (Cell Signaling, 8H10D10) (WB 1:5000)
APC-conjugated anti-ACSA-2 (Miltenyi, 130-116-245, clone REA969) (FC 1:100)
FITC-conjugated hamster anti-CD29 (Biolegend, 102205, clone HMβ1-1)(FC 1:100)

Secondary antibodies:
Alexa Fluor 488-conjugated anti-Chicken (IgG, donkey, Jackson ImmunoResearch Laboratories 703-545-155, 1:1000), Alexa Fluor 555-conjugated anti-rabbit IgG (donkey, Invitrogen, A31572, 1:1000), Alexa Fluor 647-conjugated anti-mouse IgG (goat, Invitrogen, A21236, 1:1000), Alexa Fluor 647-conjugated anti-rabbit IgG (goat, Invitrogen, A21245, 1:1000), Alexa Fluor 647-conjugated anti-goat IgG (donkey, Jackson ImmunoResearch Laboratories 705-605-147, 1:1000), Cy5-conjugated anti-rabbit IgG (donkey, Jackson ImmunoResearch, Cat#711-175-152, 1:100), Alexa Fluor 405-conjugated anti-rabbit IgG (goat, Invitrogen, A31556, 1:1000), DyLight 405-conjugated anti-mouse IgG (donkey, Jackson ImmunoResearch, Cag#715-475-151, 1:100)

IHC, immunohistochemistry; ICC, immunocytochemistry; FC, flow cytometry; WB, western blot

Validation

All antibodies with the exception of anti-Myc used in this study are commercially available, and the validation information is provided in manufacture's websites (see below). The rabbit anti-Myc antibody was generated in our laboratory (Prof K. Nagata's lab in Department of Molecular Neurobiology, Institute for Developmental Research, Aichi Developmental Disability Center), and its specificity was confirmed in original publications using Western blot (https://doi.org/10.1515/hsz-2012-0258).

chicken anti-GFP (Aves, GFP1010) (IHC 1:1000)
Format: IgY Fraction/ Concentration: 10 mg/mL/ Clonality: Polyclonal/ Isotype: IgY/
Applications: ELISA, ICC, IHC, WB/ Host Species: Chicken/ Antigen Recombinant: GFP expressed in Escherichia coli/ Antibody Registry: ID AB_2307313/ Production Notes: Chickens were immunized with purified recombinant green fluorescent protein (GFP) emulsified in Freund's adjuvant. After multiple injections, eggs were collected from the hens, and IgY fractions were prepared from the yolks and then affinity-purified antibodies were prepared using GFP conjugated to an agarose matrix. The final product is a filter-sterilized mixture of both affinity-purified antibodies (30 µg/mL) and purified IgY (10 mg/mL)./ Buffer: Sodium phosphate (10 mM, pH 7.2) buffered isotonic saline (0.9%, w/v), glycerol (50%, v/v), with sodium azide (0.02%, w/v) as an anti-microbial agent./ Dilution Ranges, WB: 1:5000-1:10000, IHC: 1:2000-1:5000, ICC: 1:2000-1:5000/ Quality Control: Antibodies were analyzed by western blot analysis (1:5000 dilution) and immunohistochemistry (1:500 dilution) using transgenic mice expressing the GFP gene product. Western blots were performed using BlokHen® (Aves Labs) as the blocking reagent, and HRP-labeled goat anti-chicken antibodies (Aves Labs, Cat. #H-1004) as the detection reagent. Immunohistochemistry used tetramethyl rhodamine-labeled anti-chicken IgY.

rabbit anti-GFP (MBL, #598) (IHC 1:1000)
Target: GFP/ Application: ChIP, EM, ICC, IHC, IP, WB/ Antibody Type: Polyclonal/ Isotype: IgG/ Immunogen: Recombinant GFP/ Host

Species: Rabbit/ Source: This antibody was purified from rabbit serum using ion exchange chromatography. The rabbit was immunized with recombinant GFP protein. The reactivity with recombinant partner and other E. coli components are absorbed using affinity chromatographic technique.

rabbit anti-RFP (Rockland, #600-401-379) (IHC 1:1000, WB 1:500)
Tested Applications: ELISA, IF, IHC, WB/ Suggested Applications, EM, FC, FISH, IP, Other/ Cross Reactivity: RFP, tdTomato/ Minimum Cross: Hu Ms and Rt Serum Proteins/ Epitope-Tag Specificity: RFP/ Host Species: Rabbit/ Clonality: Polyclonal/ Format: IgG/ Immunogen Type: Recombinant Protein/ Immunogen: The immunogen is a Red Fluorescent Protein (RFP) fusion protein corresponding to the full length amino acid sequence (234aa) derived from the mushroom polyp coral Discosoma./ Concentration: 1.06mg/mL by UV absorbance at 280 nm/ Buffer: 0.02 M Potassium Phosphate, 0.15 M Sodium Chloride, pH 7.2/ Preservative: 0.01% (w/v) Sodium Azide

mouse anti-GFAP (Sigma, G3893, clone G-A-5) (IHC 1:500, ICC 1:1000)
biological source: mouse/ antibody form: purified immunoglobulin antibody/ product type: primary antibodies/ clone: GA5, monoclonal/ species reactivity: chicken, rat, rabbit, mouse, pig, bovine, human/ manufacturer tradename: Chemicon®/ technique(s)= immunocytochemistry: suitable, immunohistochemistry (formalin-fixed, paraffin-embedded sections): suitable, western blot: suitable/ isotype: IgG1/ NCBI accession no.: NM_002055.2/ UniProt accession no.:P14136/ Specificity: Expected to cross-react with porcine, chicken, bovine, and rabbit./ The antibody reacts with GFAP from human, pig, chicken and rat. In tissue sections this antibody stains astrocytes and Bergman glia cells (Debus, E., 1983)./ Immunogen: Purified glial filament (Debus, E., 1983)./ Application: Anti-Glial Fibrillary Acidic Protein Antibody, clone GA5 is an antibody against Glial Fibrillary Acidic Protein for use in IC, IH, IH(P) & WB with more than 55 product citations./ Immunohistochemistry: 5 µg/mL/ Immunoblotting: Recognizes a 51kDa protein in reducing westerns of total brain lysates./ Format: Protein A purified. Purified mouse monoclonal IgG1 in buffer containing 0.02 M phosphate buffer, 0.25 M NaCl with 0.1% sodium azide, pH 7.6.

mouse anti-β-tubulin III (BioLegend/Covance, 801213, TuJ1) (ICC 1:1000)
Verified Reactivity: Human, Mouse, Rat/ Antibody Type: Monoclonal/ Host Species: Mouse/ Immunogen: This antibody was raised against microtubules derived from rat brain./ Formulation: Phosphate-buffered solution + 0.03% Thimerosal./ Preparation: The antibody was purified by affinity chromatography./ Concentration: 1.0 mg/ml/ Application: IHC-P, Quality tested;
WB, ICC, Verified; FC, Reported in the literature, not verified in house/ Recommended Usage: Each lot of this antibody is quality control tested by formalin-fixed paraffin-embedded immunohistochemical staining. For immunohistochemistry, a concentration range of 1.0 - 5.0 µg/ml is suggested. For Western blotting, the suggested use of this reagent is 1.0 - 5.0 µg/ml. For immunocytochemistry, a concentration range of 1.0 - 5.0 µg/ml is recommended. It is recommended that the reagent be titrated for optimal performance for each application./ Application Notes; Additional reported applications (for the relevant formats) include: flow cytometry, immunofluorescence microscopy, immunohistochemistry, Western blotting, and spatial biology (IBEX).
This antibody is well characterized and highly reactive to neuron specific Class III ß-tubulin (ßIII). TUJ1 does not identify ß-tubulin found in glial cells. TUJ1 recognizes an epitope located within the last 15 C-terminal residues.

rabbit anti-Myc (Mizutani et al. https://doi.org/10.1515/hsz-2012-0258) (WB 1:1000)
This anti-Myc antibody is originally generated in the Prof K. Nagata's lab in Department of Molecular Neurobiology, Institute for Developmental Research, Aichi Developmental Disability Center. This material should be obtained from Prof K. Nagata (knagata@inst-hsc.jp).

goat anti-Olig2 (R&D, AF2418) (IHC 1:300)
Species Reactivity: Human, Mouse, Rat/ Specificity: Detects human Olig2 in direct ELISAs and Western blots./ Source: Polyclonal Goat IgG/ Purification: Antigen Affinity-purified/ Immunogen: E. coli-derived recombinant human. Olig2 Met1-Lys323. Accession # Q13516/ Formulation: Lyophilized from a 0.2 µm filtered solution in PBS with Trehalose. See Certificate of Analysis for details. *Small pack size (-SP) is supplied either lyophilized or as a 0.2 µm filtered solution in PBS./ Applications: Western Blot: 0.1 µg/mL, Immunohistochemistry: 5-15 µg/mL.

goat anti-S100β (R&D, AF1820) (IHC 1:200)
Species Reactivity: Human/ Specificity: Detects human S100B in direct ELISAs and Western blots. In direct ELISAs, less than 1% cross-reactivity with recombinant human (rh) S100A1, rhS100A4, and rhS100A6 is observed./ Source: Polyclonal Goat IgG/ Purification: Antigen Affinity-purified/ Immunogen: E. coli-derived recombinant human S100B. Met1-Glu92. Accession # P04271/ Formulation: Lyophilized from a 0.2 µm filtered solution in PBS with Trehalose./ Applications: Immunohistochemistry 5-15 µg/mL.

goat anti-GSTπ(LifeSpan Biosciences, LS-B2376)(IHC 1:300)
Host: Goat/ Reactivity; Human, Monkey, Mouse, Rat, Bat, Bovine, Dog, Hamster, Horse, Pig (tested or 100% immunogen sequence identity)/ Clonality; Polyclonal/ Purification; Purified from goat serum by ammonium sulphate precipitation followed by antigen affinity chromatography using the immunizing peptide./ Immunogen; Peptide with sequence C-LADQGQSWKEEV, from the internal region of the protein sequence according to NP_000843.1./ Epitope: aa22-33/ Specificity; Human GSTP1./ Applications; IHC - Paraffin (5 µg/ml), Western blot (0.01 - 0.03 µg/ml) / Usage; Immunohistochemistry: LS-B2376 was validated for use in immunohistochemistry on a panel of 21 formalin-fixed, paraffin-embedded (FFPE) human tissues after heat induced antigen retrieval in pH 6.0 citrate buffer. After incubation with the primary antibody, slides were incubated with biotinylated secondary antibody, followed by alkaline phosphatase-streptavidin and chromogen. The stained slides were evaluated by a pathologist to confirm staining specificity. The optimal working concentration for LS-B2376 was determined to be 5 ug/ml./ Presentation; TBS, pH 7.3, 0.02% Sodium Azide, 0.5% BSA/ This antibody carries the LSBio 100% Guarantee.

rabbit anti-Aldh1l1 (Abcam, ab87117)(IHC 1:1000)
Host: Rabbit/ Tested applications: Suitable for WB/ Species reactivity: Reacts with Mouse, Rat. Predicted to work with Orangutan/ Immunogen: Synthetic peptide within Mouse ALDH1L1 aa 300-400 conjugated to keyhole limpet haemocyanin. The exact sequence is proprietary./ Storage buffer: pH 7.40/ Preservative: 0.02% Sodium azide/ Constituent: PBS/ Purity: Immunogen affinity purified/ Clonality: Polyclonal/
Isotype: IgG/ Concentration: 1 mg/ml/ Application: WB. Use a concentration of 1 µg/ml. Detects a band of approximately 100 kDa (predicted molecular weight: 99 kDa).

rabbit anti-Cxcr4 (Abcam, ab124824, clone UMB2) (ICC 1:300)
Host: Rabbit/ Although some customers can get this ab to work in mouse and rat successfully, we cannot reproduce this in house in IHC so cannot guarantee it./ This antibody recognizes only the non-phosphorylated C-terminus of CXCR4 (residues 341-352). Phosphorylation of S346/347 blocks antibody binding. PMID: 24154522, 25451233./ Tested applications: Suitable for IHC (Frozen), Flow Cyt (Intracellular), WB, IHC (Paraffin), ICC/ Species reactivity: Reacts with Mouse, Rat, Human/ Immunogen: Synthetic peptide within Human CXCR4 aa 300 to the C-terminus (C terminal). The exact sequence is proprietary./ Storage buffer: pH 7.20/ Preservative: 0.01% Sodium azide/ Constituents: 40% Glycerol, 0.05% BSA, 59% PBS/ Concentration: 2.709 mg/ml/ Purity: Protein A purified/ Clonality: Monoclonal/Clone number: UMB2/ Isotype: IgG

mouse anti-β-Actin (Cell Signaling, 8H10D10) (WB 1:5000)
Endogenous MW (kDa): 45/ Isotype: Mouse IgG2b/ Application: Western Blotting 1:1000, Immunohistochemistry (Paraffin) 1:8000 - 1:32000, Immunofluorescence (Immunocytochemistry) 1:2500 - 1:10000, Flow Cytometry (Fixed/Permeabilized) 1:200 - 1:800/ Storage: Supplied in 10 mM sodium HEPES (pH 7.5), 150 mM NaCl, 100 µg/ml BSA, 50% glycerol and less than 0.02% sodium azide/ Specificity: β-Actin (8H10D10) Mouse mAb detects endogenous levels of total β-actin protein. Due to the high sequence identity between the cytoplasmic actin isoforms, β-actin and cytoplasmic γ-actin, this antibody may cross-react with cytoplasmic γ-actin. It does not cross-react with α-skeletal, α-cardiac, α-vascular smooth, or γ-enteric smooth muscle isoforms./ Species Reactivity: Human, Mouse, Rat, Hamster, Monkey, Dog/ Source, Purification: Monoclonal antibody is produced by immunizing animals with a synthetic peptide corresponding to amino-terminal residues of human β-actin.

APC-conjugated anti-ACSA-2 (Miltenyi, 130-116-245, clone REA969) (FC 1:100)
Clone:REA969/Type of antibody:Primary antibodies, Recombinant antibodies/Isotype:recombinant human IgG1/Applications:FC, MICS, IF, IHC/Alternative names:ACSA2/Conjugate:APC/Recommended dilution:1:50/Tested:QC tested

FITC-conjugated hamster anti-CD29 (Biolegend, 102205, clone HMβ1-1)(FC 1:100)
Clone:HMβ1-1 (See other available formats)/Other Names:integrin β1, VLA-β chain, β1 integrin, GPIIa, ITGB1/Isotype:Armenian Hamster IgG/Description:CD29 is a 130 kD protein, also known as integrin β1, VLA-β chain, or GPIIa. It is a member of the integrin family, expressed broadly on leukocytes, endothelial cells, smooth muscle, and epithelial cells. In association with CD49a-f, CD29 forms the VLA-1 through VLA-6 complexes, respectively. It plays an important role in cell-cell or cell-matrix interaction. The HMß1-1 antibody reacts with both mouse and rat CD29. It is able to block cell adhesion and inhibit T cell proliferation./Verified Reactivity:Mouse, Rat/Antibody Type:Monoclonal/Immunogen:Purified mouse VLA-4 (α4β1, CD49d/CD29)/Formulation:Phosphate-buffered solution, pH 7.2, containing 0.09% sodium azide./Preparation:The antibody was purified by affinity chromatography, and conjugated with FITC under optimal conditions./Concentration:0.5 mg/ml/Application:FC - Quality tested/Recommended Usage:Each lot of this antibody is quality control tested by immunofluorescent staining with flow cytometric analysis. For flow cytometric staining, the suggested use of this reagent is ≤ 1.0 µg per 106 cells in 100 µl volume. It is recommended that the reagent be titrated for optimal performance for each application.

Eukaryotic cell lines

Policy information about cell lines and Sex and Gender in Research

Cell line source(s)	COS-7 (#CRL-1651) and Neuro2a (#CCL-131) cells were obtained from ATCC.
Authentication	Not performed
Mycoplasma contamination	Not performed
Commonly misidentified lines (See ICLAC register)	Not used.

Animals and other research organisms

Policy information about studies involving animals; ARRIVE guidelines recommended for reporting animal research, and Sex and Gender in Research

Laboratory animals	Timed pregnant ICR mice (Japan SLC, Shizuoka, Japan) were purchased. Olig2-CreER and Z/EG mice (both 8-24 weeks) were maintained and bred at Keio University School of Medicine. Flt1-DsRed mice were maintained by crossing heterozygote males with wild type ICR females (both 8-24 weeks) at Institute for Developmental Research. Aldh1l1-GFP mice (MMRRC, Stock #011015) were obtained from the University of California at Davis. Heterozygous male mice were mated with C57B/6J females (both 8-24 weeks) at Keio University School of Medicine. The stages of manipulation and sampling were indicated in the corresponding Figure legends (embryonic day 15 to postnatal day 30). All mice were housed at 22-24C̄ with 40-60% humidity under the 12 h light/12 h dark cycle.
Wild animals	No wild animals were used in the study.
Reporting on sex	We have not experienced any sexual difference in migration and positioning of astrocytes.<br>We mixed the data from male and female mice.
Field-collected samples	No filed-collected samples was used.
Ethics oversight	All protocols for animal handling and treatments conducted in Institute for Developmental Research were approved by the Animal Care and Use Committee of Institute for Developmental Research, Aichi Developmental Disability Center (approval number: 2019-013). Those in Keio University were approved by the Keio University Institutional Animal Care and Use Committee in accordance with the Institutional Guidelines on Animal Experimentation at Keio University (approval number: A2021-030).

Note that full information on the approval of the study protocol must also be provided in the manuscript.
